# Some historical remarks on the conservative treatment of facial paralysis – comment on 'Temporary ectropion therapy by adhesive taping: a case study'

**DOI:** 10.1186/1746-160X-4-26

**Published:** 2008-11-19

**Authors:** Robert C van de Graaf

**Affiliations:** 1 C.F. Von Graefe Institute, Zijlsterried 32, 9746 PB Groningen, the Netherlands

Dear Editor,

In their recent article entitled 'Temporary ectropion therapy by adhesive taping: a case study', Drs Schrom and Habermann 'propose a simple method to correct temporary ectropion in facial palsy by applying an adhesive strip' [1]. The authors seem to believe that their method had not been used previously as they state: 'Adhesive strips have only been used in individual cases to correct lagophthalmos, entropion or ptosis of the eyebrow' [1]. This statement appears to be based on several medical journal articles, but none of the contemporary textbooks on facial paralysis is listed in their reference list [1]. Had the authors, however, studied 'The Facial Palsies', they would have read: 'Ectropion of the lower eyelid may be alleviated by taping it upwards and laterally, e.g. with a steristrip. These non-surgical resources may certainly help in the acute phase. They may even be preferred by patients with temporary facial paralysis, e.g. the Bell's palsies' [2]. In the other classic on facial paralysis 'The Facial Nerve' the authors could have read: 'To support a drooping lower lid, the end of the tape should be applied to the center of the lower lid with the upper edge about 1/8 in below the lashes. The tape should then be pulled up laterally and secured to the lateral orbital rim. Trial and error will demonstrate the best way of eliminating lid droop in a given patient. Normalization of lower lid position will bring the reservoir of tears into contact with the cornea and will decrease the palpebral aperture, thus limiting abnormal evaporation of tears and reducing irritation of the palpebral conjunctiva caused by ectropion' [3]. However, the use of adhesive strips for correcting paralytic ectropion has not only been discussed in books. In a paper entitled: 'Simple measures for acute peripheral facial paralysis' the following statement can be read: 'The lower end of the strip of tape is first fixed as high as possible in the middle of the lower eyelid. It is necessary to fix it as high as possible, under the eyelash, since otherwise the eyelid curls up and the uncovered distance between the eyelids does not become narrower. The skin at the lateral corner of the eye is then folded, by means of traction on the strip of tape, and the lower eyelid is pulled up and sideways by fixation of the tape temporally. Besides a good cosmetic effect, this method gives some protection to the cornea. In many patients suffering from facial paralysis the cornea may be sufficiently protected in this way. These simple measures, suggested by Professor Jongkees, have been used for many years with success' [4]. We have to realise that the use of adhesive strips as a conservative method to treat facial paralysis was already described in the early nineteenth century [5,6], when most of the current surgical techniques had not yet been developed.
